# Effects of *Tithonia diversifolia* (Hemsl.) A. Gray Extract on Adipocyte Differentiation of Human Mesenchymal Stem Cells

**DOI:** 10.1371/journal.pone.0122320

**Published:** 2015-04-07

**Authors:** Claudia Di Giacomo, Luca Vanella, Valeria Sorrenti, Rosa Santangelo, Ignazio Barbagallo, Giovanna Calabrese, Carlo Genovese, Silvana Mastrojeni, Salvatore Ragusa, Rosaria Acquaviva

**Affiliations:** 1 Dept. of Drug Science—Biochemistry Section, University of Catania, Catania, Italy; 2 IOM Ricerca SRL, Via Penninazzo, 11, Viagrande, Catania, Italy; 3 Dept. of Bio-Medical Sciences, Section of Microbiology, University of Catania, Catania, Italy; 4 Dept. of Health Sciences, University “Magna Graecia” of Catanzaro, Catanzaro, Italy; University College London, UNITED KINGDOM

## Abstract

*Tithonia diversifolia* (Hemsl.) A. Gray (Asteraceae) is widely used in traditional medicine. There is increasing interest on the *in vivo* protective effects of natural compounds contained in plants against oxidative damage caused from reactive oxygen species. In the present study the total phenolic and flavonoid contents of aqueous, methanol and dichloromethane extracts of leaves of *Tithonia diversifolia* (Hemsl.) A. Gray were determined; furthermore, free radical scavenging capacity of each extract and the ability of these extracts to inhibit *in vitro* plasma lipid peroxidation were also evaluated. Since oxidative stress may be involved in trasformation of pre-adipocytes into adipocytes, to test the hypothesis that *Tithonia* extract may also affect adipocyte differentiation, human mesenchymal stem cell cultures were treated with *Tithonia diversifolia* aqueous extract and cell viability, free radical levels, Oil-Red O staining and western bolt analysis for heme oxygenase and 5'-adenosine monophoshate-activated protein kinase were carried out. Results obtained in the present study provide evidence that *Tithonia diversifolia* (Hemsl.) A. Gray exhibits interesting health promoting properties, resulting both from its free radical scavenger capacity and also by induction of protective cellular systems involved in cellular stress defenses and in adipogenesis of mesenchymal cells.

## Introduction

The genus *Tithonia* comprises 13 taxa, which are distributed in eleven species. *Tithonia diversifolia* (Hemsl.) A. Gray (Asteraceae) is native to Mexico and also grows in parts of Africa, Australia, Asia, and other countries of North America. *Tithonia diversifolia* (Hemsl.) A. Gray and its extracts are traditionally used for the treatment of diabetes, diarrhea, menstrual pain, malaria, hematomas, hepatitis, hepatomas, and wound healing [1, 2]. Recently it has been suggested that these effects might be ascribed to terpenoids and flavonoids contained in the aerial parts of *Tithonia diversifolia* [3, 4]. Several studies investigated anti-inflammatory, analgesic, antimalarial, antimicrobial and antidiabetic activities; although these investigations revealed the potential of this plant and its constituents for different pharmacological/therapeutic activities [4], studies are needed in order to understand the molecular modes of action of *Tithonia* and its extracts.

Recently, intense interest has focused on the antioxidant properties of natural products. In particular, natural products that may act by preventing the free radical generation, neutralizing free radicals by non-enzymatic mechanisms and/or by enhancing the activity of endogenous antioxidants [5] such as stress-inducible proteins. Heme oxygenase (HO) (EC 1.14.99.3) is a microsomal enzyme that oxidatively cleaves heme and produces biliverdin, carbon monoxide (CO) and iron [6]. To date, two isoforms of HO have been identified: HO-1, or inducible enzyme, and HO-2 or constitutive isoform [6–10]. A substantial body of evidence demonstrates that HO-1 induction represents an essential step in cellular adaptation to stress subsequent to pathological events [11–14]; then HO-1 hyper-expression can be considered both a marker of cellular stress and also regarded as a potential therapeutic target in a variety of oxidant-mediated diseases [15].

Recently it has been reported that polyphenolic natural compounds are able to induce potently HO-1 expression, exercising protective effects. As a consequence, the beneficial actions attributed to several natural substances could be also due to their intrinsic ability to activate the HO-1 pathway [16–18].

Adipocytes play a central role in regulating adipose mass and obesity. The increased adipose mass in obesity is caused both by adipose tissue hypertrophy, and also by adipose tissue hyperplasia, which affects the transformation of pre-adipocytes into adipocytes [19, 20]. Thus, adipocyte differentiation and the amount of fat accumulation are associated with the development of obesity.

Obesity was correlated to high levels of lipid peroxidation and/or decreased antioxidant capacities [21, 22]. When free-radical formation exceeds protective antioxidant mechanisms, or the later are compromised, oxidative stress occurs; increasing evidence from research show that oxidative stress is associated with the pathogenesis of obesity and it has been demonstrated that *in vitro* pre-adipocyte proliferation and differentiation can be controlled by redox metabolism [23–26] suggesting that reactive oxygen species (ROS) may be involved in adipocyte differentiation.

The 5'-adenosine monophoshate-activated protein kinase (AMPK) is a heterotrimeric protein consisting of one catalytic subunit (α) and two non-catalytic subunits (β and γ). As AMP binds to the γ-subunit, it undergoes a conformational change which exposes the catalytic domain found in the α subunit; AMPK becomes activated (pAMPK) when phosphorilation occurs at the threonine residue within the kinase domain [27, 28]. AMPK has been proposed to act as a main metabolic switch in response to changes in cellular meabolism [29]. When activated, AMPK leads to an inhibition of energy-consuming biosynthetic pathways and concomitant activation of catabolic ATP-producing pathways [30, 31]. AMPK also acts as a fuel sensor in regulating glucose and lipid homeostasis in adipocytes by many additional effects both on genes and specific enzymes [32, 33].

In the present study the free radical scavenging capacity of different concentrations of aqueous, methanolic and dichloromethane leaf extracts of *Tithonia diversifolia* (Hemsl.) A. Gray was evaluated by *in vitro* assays; moreover the ability of *Tithonia* extract to inhibit plasma lipid peroxidation in a cell-free system was also tested.

In order to test the hypothesis that *Tithonia* leaf extract may also affect adipocyte differentiation, human mesenchymal stem cells (hMSC) were cultured in the presence or absence of *Tithonia diversifolia* (Hemsl.) A. Gray extracts and adipogenesis was measured by Oil-Red O staining. In the same cell cultures ROS levels were determined, and HO-1 and pAMPK expressions were also evaluated by western blot analysis.

## Material and Methods

### Ethics Statement

According to Italian law, we have to ask the opinion of the Ethics Committee only in the case of clinical trials; if blood, tissues or cells from donors or patients are used for research purposes, it is necessary that donors/patients give their written consent, not subject to the opinion of the Ethics Committee. The donors/patients are anonymous, because their generality are known only to the doctor who took the sample and that keeps their written consent. However in order to avoid any quandary we obtained the consent by Ethics Committee (N° IRB IOM 07 2012 of 08 February 2012).

### Chemicals

Water, methanol and dichloromethane used for the extractions were of analytical grade and were purchased from Merck S.p.A. (Milano, Italy); all the other solvent, chemicals and reference compounds were purchased from Sigma-Aldrich (Milano, Italy).

### Plant collection and preparation of extracts

The leaves of *Tithonia diversifolia* (Hemsl.) A. Gray were collected in August 2010 in the Eastern Democratic Republic of Congo and kindly provided by Sister Kavira Muhyama, Diocese of Butembo, Bunyuka (GPS Coordinates: Lat. 0.127752°; Long. 29.287371. B.P., 179 Butembo, Nord-Kivu). The field studies did not involve endangered or protected species. Sister Kavira Muhyama issued the permit for each location.

A voucher specimen of the plant was deposited in the herbarium of Department of Health Sciences, University “Magna Graecia” of Catanzaro.

Crude methanolic and dichloromethane extracts were obtained by maceration of 5 g of each powdered plant sample three times in 50 mL of solvent, for 45 min under constant shaking at room temperature. For the aqueous extracts, 100 mL of distilled water was used to extract 2 g of powdered plant material and the mixture obtained was boiled for 1 h. The extracts were filtered and evaporated to dryness under reduced pressure with a rotatory evaporator.

### Total phenolic and flavonoid content

The concentration of total phenolic compounds was determined spectrophotometrically, using the Folin–Ciocalteu total phenols procedure, described by Ballard et al. [34], with modifications. Known amounts of Gallic acid were used to prepare the standard curve. Appropriately diluted (3.5%, w/v) test extracts (0.1 mL) and the gallic acid standard solutions (0.1 mL) were transferred to 15 mL test tubes. 3.0 mL of 0.2 N Folin–Ciocalteu reagent were added to each test tube and mixed using a vortex mixer. After 1 min, 2.0 mL of 9.0% (w/v) Na_2_CO_3_ in water were added and the solution was mixed. Absorbance was determined at λ = 765 nm. The concentration of total phenolic compounds in the extracts was determined comparing the absorbance of the extract samples to that of the gallic acid standard solutions. All samples were determined in triplicate. Total phenolic content was expressed as μMoles gallic acid/L ± S.D. Results represent the mean ± S.D. of 5 determinations.

The flavonoid concentration was measured using a colorimetric assay [35], with modifications. A standard curve of cathechin was used for quantification. Briefly, 25 mL of aqueous, methanolic and dichloromethane extracts and/or cathechin standard solutions were added to 100 mL of H_2_O. At time zero, 7.5 mL of 5% NaNO_2_ were added; after 5 min, 7.5 mL of 10% AlCl_3_ were added and at 6 min, 50 mL of 1 M NaOH were added. Each reaction mixture was then immediately diluted with 60 mL of H_2_O and mixed. Absorbances of the mixtures upon the development of pink color were determined a λ = 510 nm relative to a prepared blank. The total flavonoid contents of the samples are expressed as μMoles catechin/L. Each result represents the mean ± S.D. of 5 experimental determinations.

### Scavenger effect on superoxide anion (SOD-like activity)

Superoxide anion was generated *in vitro* as described by Acquaviva et al. 2012 [36]. A total volume of 1 mL of the assay mixture contained: 100 mM triethanolamine-diethanolamine buffer, pH 7.4, 3 mM NADH, 25 mM/12.5 mM EDTA/MnCl_2_, 10 mM β-mercapto-ethanol; samples contained different concentrations of the three (aqueous, methanolic and dichloromethane) extracts of leaves of *Tithonia diversifolia* (Hemsl.) A. Gray. After 20 min incubation at 25°C, the decrease in absorbance at λ = 340 nm was measured. Results are expressed as percentage of inhibition of NADH oxidation. SOD (80 mU) was used as reference compound. Each result represents the mean ± S.D. of 5 experimental determinations.

### Determination of lipid hydroperoxide levels in the plasma of a healthy donor

Heparinized venous blood of a healthy volunteer donor (male, 27 years old), who agreed to take part in the study and gave his written consent. Since this is a non-therapeutic trial it was carried out with the consent of the subjects legally acceptable according our Italian Government (Legge 675/1996 and DL 196/2003, art. 40. Art 32 Codice Italiano di Deontologia Medica).

Heparinized venous blood was collected after overnight fasting. Plasma was separated by centrifugation at 800 *g* for 20 min. Plasmatic lipid hydroperoxide levels were evaluated by oxidation of Fe^2+^ to Fe^3+^ in the presence of xylenol orange at λ = 560 nm [37]. Plasma aliquots (500 μL) were diluted 1:1 with oxygenated PBS and incubated at 37°C for 2 h with or without different concentrations of the aqueous extracts in a total volume of 1 mL. Results are expressed as percentage of inhibition respect to control (plasma incubated in absence of test compounds) and represent the mean ± S.D. of 5 experimental determinations.

### Isolation and adipogenic differentiation of human adipose MSCs

Adipose tissue sample was obtained from a patient underwent abdominal plastic surgery (male, 30 years old, 98 kg b.w.); the subject provided his written consent before inclusion in the study. Since this is a non-therapeutic trial, it was carried out with the consent of the subject legally acceptable according our Italian Government (Legge 675/1996 and DL 196/2003, art. 40. Art 32 Codice Italiano di Deontologia Medica).

Adipose tissue sample was removed under sterile conditions, washed in PBS, minced, and digested with 1 mg/mL collagenase type I in 0.1% BSA for 1 h at 37°C in a shaking water bath. The pellet was collected by centrifugation at 650 *g* for 10 min and then treated with red blood cell lysis buffer (155 mM NH_4_Cl, 10 mM KHCO_3_ and 0.1 mM EDTA) for 10 min at room temperature. After centrifugation, the cellular pellet was filtered through a 100-μm mesh filter to remove debris. The filtrate was centrifuged, and the obtained stromal vascular fraction was plated onto 100 mm cell culture dishes in complete culture medium (DMEM containing 20% fetal bovine serum, 100 μg/mL streptomycin, 100 U/mL penicillin, 2 mM L-glutamine, and 1 μg/mL amphotericin-B). Cells were cultured at 37°C in humidified atmosphere with 5% CO2. After 24 h, non-adherent cells were removed, and adherent cells were washed twice with PBS. Confluent cells were trypsinized and expanded in T75 flasks (passage 1). A confluent and homogeneous fibroblast-like cell population was obtained after 2–3 weeks of culture. For all the experiments, only cells at early passages were used. At 50–60% confluence the medium was replaced with adipogenic medium, and the cells were cultured for additional 14 days. The adipogenic media consisted of complete culture medium supplemented with DMEM-high glucose (4.5 g/L), 10% (w/v) fetal bovine serum (FBS), 10 mg/mL insulin, 0.5 mM dexamethasone and 0.1 mM indomethacin. During adipogenic differentiation some flasks were added with different concentrations of the *Tithonia* extract. Media were changed every 2 days.

### Cell viability assay

Cell viability was determined by the MTT assay, measuring the activity of cellular enzymes that reduce the tetrazolium dye, 3-(4,5-dimethylthiazol-2-yl)-2,5-diphenyltetrazolium bromide, a yellow tetrazole (MTT), to its insoluble formazan, giving a purple color in living cells [38]. The cells were plated at 8x10^3^ cells per well of a 96-multiwell flat-bottomed 200 μl microplate. The optical density of each well sample was measured with a microplate spectrophotometer reader (Synergy HT multi-mode microplate reader, BioTek, Milano, Italy) at λ = 570 nm. Results are expressed as percentage of cell viability; each result represents the mean ± S.D. of 5 experimental determinations.

### Oil Red O staining

Oil Red O staining is an assay performed to stain mature adipocytes in adipogenic-induced cultures. The mechanism of the staining of lipids is a function of the physical properties of the dye, which more soluble in the lipid than in the vehicular solvent. For Oil Red O staining, 0.21% Oil Red O in 100% isopropanol was used. Briefly, MSC-derived adipocytes, after 14 days, were fixed in 10% formaldehyde, washed in Oil-red O for 10 min, rinsed with 60% isopropanol, the Oil red O eluted by adding 100% isopropanol for 10 min and Optical Density measured at λ = 490 nm in a microplate reader (Synergy HT multi-mode microplate reader, BioTek, Milano, Italy). Results are expressed as optical density (OD) at λ = 490 nm. Each result represents the mean ± S.D. of 5 experimental determinations.

### Determination of ROS

Determination of ROS was performed by using the fluorescent probe 2',7'-dichlorofluorescein diacetate, DCFH-DA, as previously described [39]. Briefly, 100 μL of 100 μM DCFH-DA, dissolved in 100% methanol, was added to the cellular medium, and cells were incubated at 37°C for 30 minutes. After incubation, cells were lysed and centrifuged at 10,000 x *g* for 10 min. The fluorescence (corresponding to oxidized CDF) was monitored spectrofluorometrically (λ_ex_ = 488 nm; λ_em_ = 525 nm), using an F-2000 spectrofluorimeter (Hitachi) and results were expressed as Fluorescence Intensity (F.I.) x 1 x10^6^/mg protein. Total protein content in each sample was evaluated according to Lowry et al. [40].

### Western blot analysis

hMSCs cells were washed with PBS and then trypsinized (0.05% trypsin w/v with 0.02% EDTA). The pellets were lysed in buffer (50 mM Tris-HCl, 10 mM EDTA, 1% v/v Triton X-100, 1% phenylmethylsulfonyl fluoride (PMSF), 0.05 mM pepstatin A and 0.2 mM leupeptin) and, after mixing with sample loading buffer (50 mM Tris-HCl, 10% w/v SDS, 10% v/v glycerol, 10% v/v 2-mercaptoethanol and 0.04% bromophenol blue) at a ratio of 4:1, were boiled for 5 min. Samples (20 μg protein) were loaded into 8 or 12% SDS-polyacrylamide (SDS-PAGE) gels and subjected to electrophoresis (120 V, 90 min). The separated proteins were transferred to nitrocellulose membranes (Bio-Rad, Hercules, CA, USA; 1 h, 200 mA per gel). After transfer, the blots were incubated with Li-Cor Blocking Buffer for 1 h, followed by overnight incubation with 1:1000 dilution of the primary antibody. Primary polyclonal antibodies directed against HO-1, was purchased from Enzo Life Sciences (Farmingdale, NY, USA) while pAMPK was purchased from Cell Signaling Technology (Danvers, MA, USA). After washing with TBS, the blots were incubated for 1 h with secondary antibody (1:1,000). Protein detection was carried out using a secondary infrared fluorescent dye conjugated antibody absorbing at λ = 800 nm or λ = 700 nm. The blots were visualized using an Odyssey Infrared Imaging Scanner (Li-Cor Science Tec) and quantified by densitometric analysis performed after normalization with β-actin (Santa Cruz Biotechnology, Santa Cruz, CA, USA). Results were expressed as arbitrary units (AU).

### Statistical Analysis

One-way analysis of variance (ANOVA) followed by Bonferroni’s *t* test was performed in order to estimate significant differences among samples. Data were reported as mean values ± S.D. and differences between groups were considered to be significant at p<0.005.

## Results and Discussion

Table 1 reports the total phenolic and flavonoid contents in the three different *Tithonia* extracts. Aqueous extract resulted richer in phenolic and flavonoid compounds compared to methanol and dichlorometane extracts.

**Table 1:** Total polyphenols and total flavonoids in three different extracts of leaves of *Tithonia diversifolia* (Hemsl.) A. Gray.

Extract	Total Phenolic content μM Gallic acid	Total Flavonoid Content μM Catechin
Aqueous	52 ± 0.08	59 ± 0.09
Methanolic	31 ± 0.09*	34 ± 0.07*
Dichloromethane	29 ± 0.02*	39 ± 0.06**

* = *p*< 0.001 vs aqueous extract;

** = *p*< 0.005 vs aqueous extract.

**Fig 1:**
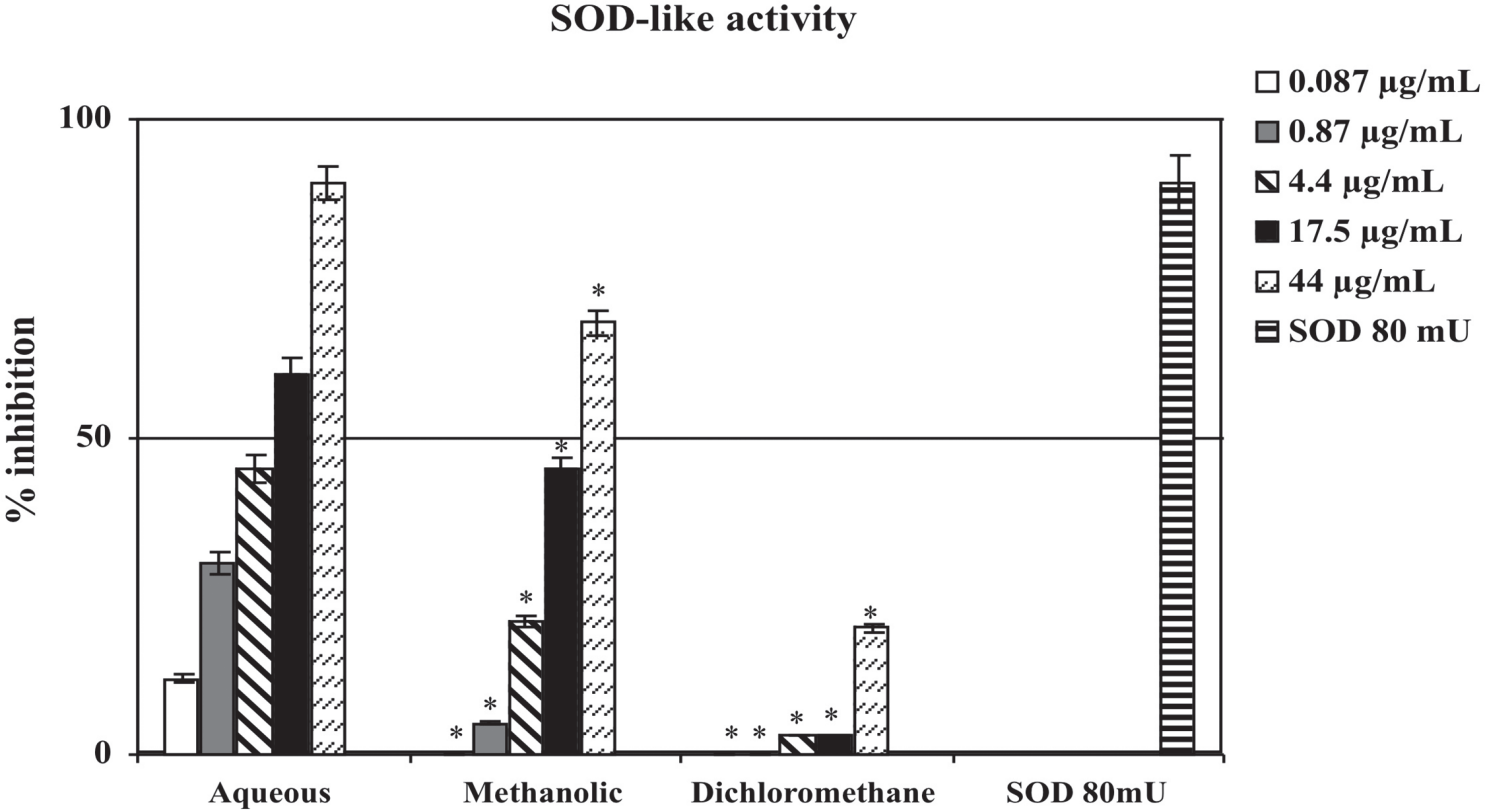
Scavenger effect of extracts of *Tithonia diversifolia* (Hemsl.) A. Gray on superoxide anion; results are expressed as percentage of inhibition of NADH oxidation (rate of superoxide anion production was 4 nmoles/min). Each value represents the mean ± SD of 5 experimental determinations. * = p<0.001 *vs*. the same concentration of the aqueous extract.

Consistent with their different polyphenol contents, scavenger activities of the three extracts differed depending on the type of extract. The aqueous extract has proven the most effective scavenger, with an effect that, at 0.044 μg/mL, was comparable with 80 mU superoxide dismutase (SOD) (Fig 1). The methanolic and dichloromethane extracts exhibited scavenger activities lower than aqueous; the less active was dichloromethane extract (Fig 1).

Based on results regarding phenolic and flavonoid contents, and taking into account findings about scavenger activities, aqueous extract was used for subsequent experiments.

**Fig 2:**
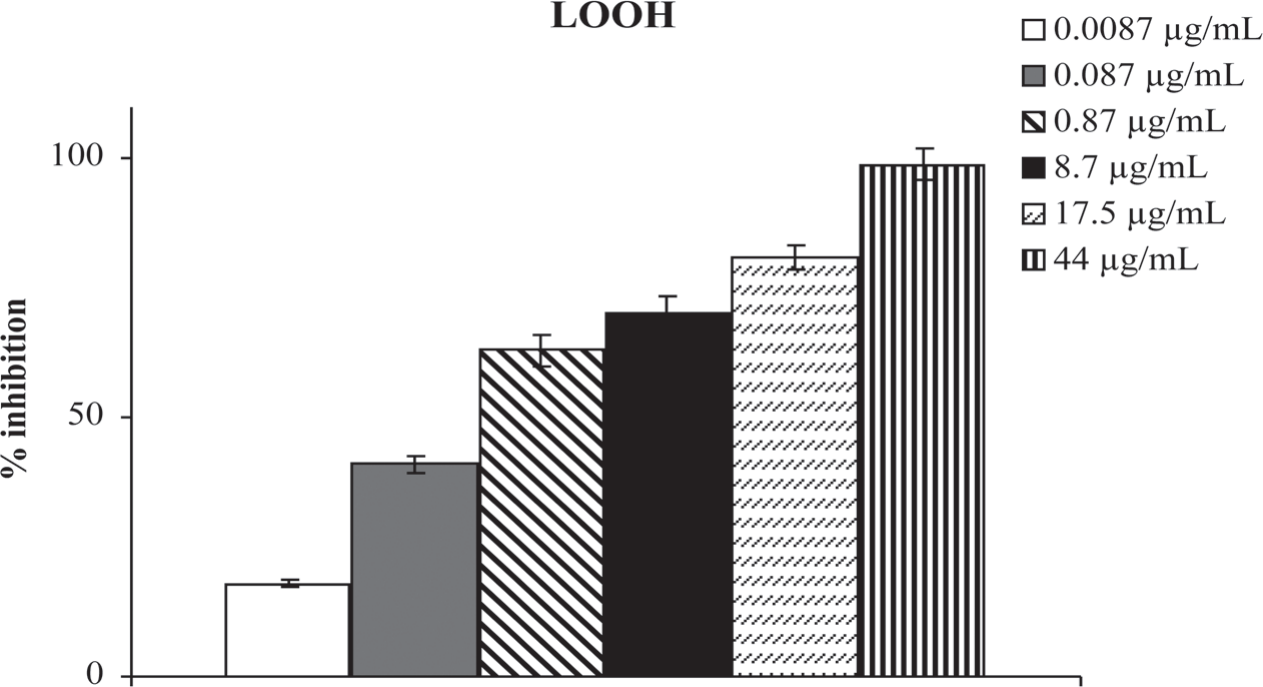
Effect of aqueous extract of *Tithonia diversifolia* (Hemsl.) A. Gray on LOOH production in plasma; results are expressed as percentage of inhibition of LOOH formation with respect to the same sample incubated in absence of the extract. Each value represents the mean ± SD of 5 experimental determinations.

Fig 2 shows results obtained by incubating plasma of a healthy donor in the presence of different concentrations of aqueous extract of *Tithonia*; as seen, the presence of the extract during incubation caused a significant and dose-dependent inhibition of plasma lipid hydroperoxide formation.

These results demonstrated that antioxidants present in aqueous extract of leaves of *Tithonia diversifolia* (Hemsl.) A. Gray are able to counteract radical chain reactions, preventing peroxidative damage of plasma lipids beyond the action of antioxidants naturally present in plasma.

In the present study we also tested the hypothesis that the aqueous extract of *Tithonia diversifolia* (Hemsl.) A. Gray might affect adipogenic differentiation of hMSCs. These cells are capable of differentiating into many different phenotypes, including osteoblasts, myocytes, chondrocytes and adipocytes *in vivo* and *in vitro* [41–43]. The directed differentiation of MSCs can be performed *in vitro* using the appropriate media and the adipogenic differentiation is confirmed by specific staining.

**Fig 3:**
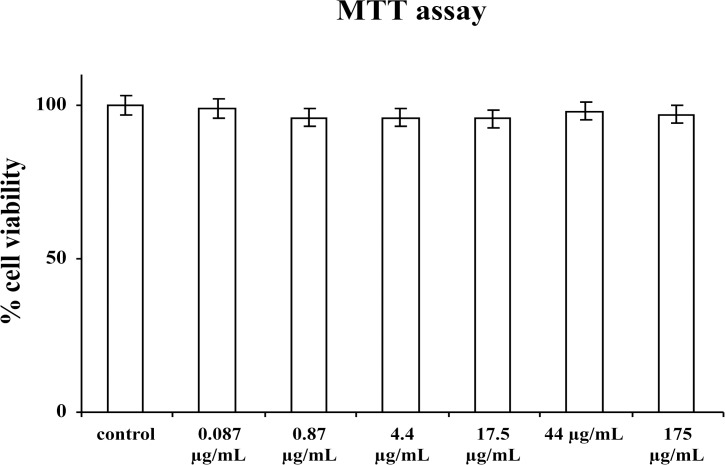
Cell viability in hMSC cultures exposed to different concentrations of aqueous extract of leaves of *Tithonia diversifolia* (Hemsl.) A. Gray; values represent the mean ± SD of 5 experiments.

As seen in Fig 3, none of the concentrations used resulted toxic for hMSC cultures: in fact, no significant difference was observed by MTT test in hMSC cultures exposed to different concentrations of aqueous extract of *Tithonia diversifolia* (Hemsl.) A. Gray with respect to untreated control (MSCs cultured in the absence of extract).

**Fig 4:**
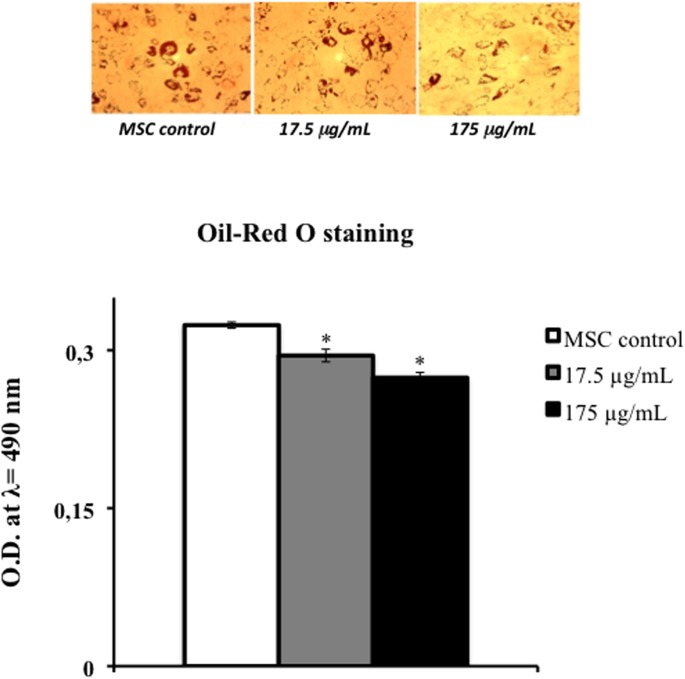
Effect of aqueous extract of leaves of *Tithonia diversifolia* (Hemsl.) A. Gray on adipogenesis of hMSCs as measured by Oil-Red O staining. Each value represents the mean ± S.D. of 5 experimental determinations. * = p< 0.001 *vs* control.

Fig 4 reports data regarding Oil Red O staining; our results demonstrated increased adipogenesis and accumulation of lipid droplets in cultured human adipose tissue-derived mesenchimal cells compared to the same cells treated with the aqueous extract of *Tithonia diversifolia* (Hemsl.) A. Gray.

Similar results were also demonstrated with other herbal extract such as *Momordica foetida* Schumach. et Thonn. [44].

**Fig 5:**
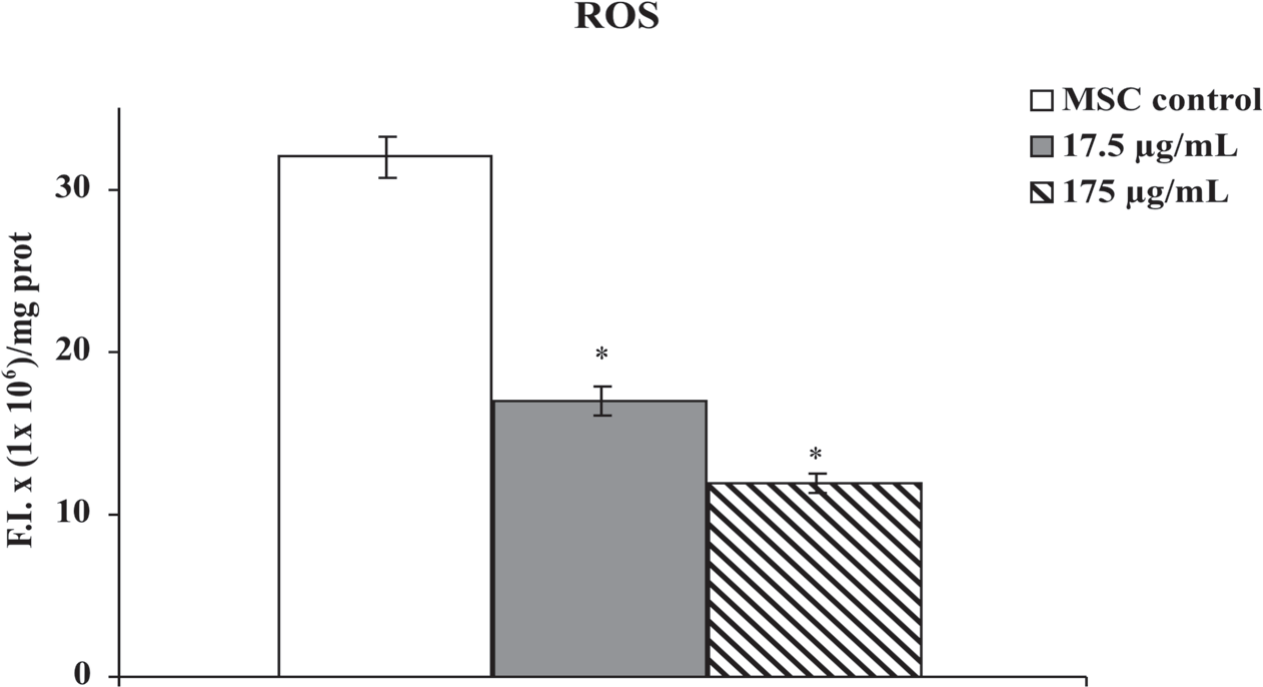
Determination of ROS by DCFH method in cultured hMSC: effect of aqueous extract of *Tithonia diversifolia* (Hemsl.) A. Gray. Each value represents the mean ± S.D. of 5 experimental determinations. * = p< 0.001 *vs* the same cells cultured in the absence of plant extract (MSC control). Results are expressed as Fluorescence Intensity (F.I.) x 1 x 10^6^/mg prot).

It has been suggested that increased levels of ROS, with consequent shifting of the intracellular redox status *vs* oxidant conditions, promote adipogenesis [24, 26, 45, 46]. Then, the decreased adipogenesis observed in hMSCs cultured in the presence of aqueous extract of *Tithonia diversifolia* (Hemsl.) A. Gray might be ascribed to its free radical scavenger effects; in order to verify this hypothesis, ROS were determined in hMSC cultures using the fluorescent probe DCFH-DA. After diffusion into the cells, DCFH-DA is deacetylated by cellular esterases to a non-fluorescent compound that can be oxidized by ROS into a highly fluorescent compound, 2',7'-dichlorofluorescein (DCF), whose fluorescence intensity is proportional to the levels of ROS [47]. As reported in Fig 5, the exposure for 72h of hMSCs to 17.5 μg/mL or 175 μg/mL aqueous extract of leaves of *Tithonia diversifolia* (Hemsl.) A. Gray resulted in a significant decrease in ROS. These data confirmed results obtained using *in vitro* cell-free systems and further support our hypothesis that antiadipogenic activity of *Tithonia diversifolia* (Hemsl.) A. Gray might be due to a decrease in intracellular ROS levels.

HO-1 (also known as stress protein HSP32) can be over-expressed in many tissues following stressful stimuli and a wide range of conditions characterized by alteration of the cellular redox state [11, 17, 48–53]. HO-1 expression might represent an important protective endogenous mechanism; in this regard, there are several reports about the beneficial effects of the induction of this enzyme in several pathological conditions [54–57]. In this context, pharmacologic modulation of HO-1 system may represent an effective strategy in several pathologic conditions but it is important to induce HO-1 expression without causing cell damages. Recently the ability of several natural antioxidants to induce HO-1 has been reported [16, 58–62].

**Fig 6:**
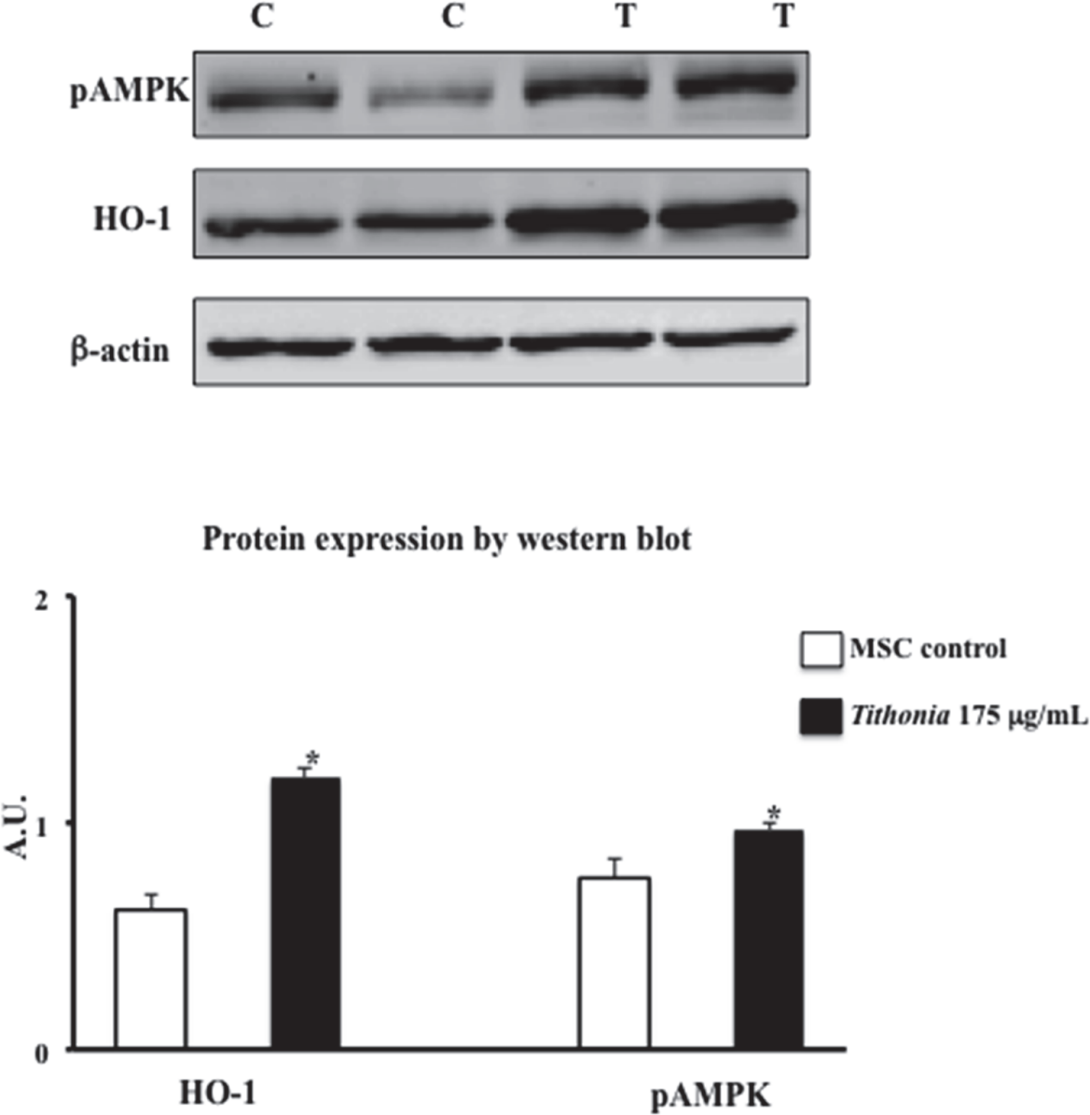
Representative Western bloting of HO-1 and pAMPK: effect of aqueous extract of *Tithonia diversifolia* (Hemsl.) A. Gray on HO-1 and pAMPK expression in cultured hMSCs. Results, expressed as arbitrary units (A.U.), represent the mean ± S.D. of 5 experimental determinations. * = p< 0.005 with respect to tcontrol (C = hMSC control, T = hMSCs treated with 175 μmg/ml of aqueous extract of *Tithonia diversifolia* (Hemsl.) A. Gray).

In order to test the hypothesis that antioxidant acticity of *Tithonia diversifolia* (Hemsl.) A. Gray may be also mediated by the induction of HO-1, in the present study HO-1 expression was determined by western blot analysis in hMSCs exposed for 72 h to 175 μg/mL *Tithonia diversifolia* (Hemsl.) A. Gray aqueous extract. Results reported in Fig 6 demonstrated that the presence of the aqueous extract of *Tithonia diversifolia* (Hemsl.) A. Gray caused a significant increase in HO-1 expression. These results confirmed that antioxidant effect of *Tithonia diversifolia* (Hemsl.) A. Gray is not merely due to a direct free-radical scavenger activity, but it is also mediated by an induction of protective cellular systems such as HO-1.

We also examined whether AMPK activation might be involved in the inhibition of adipocyte differentiation by aqueous extract of *Tithonia diversifolia* (Hemsl.) A. Gray; then, hMSCs were exposed to the extract and pAMPK was measured by western blot analysis, using phosphorylated antibody. The results (Fig 6) showed that phosphorylated AMPK levels were significantly increased in hMSC cultures exposed to 175 μg/mL *Tithonia diversifolia* (Hemsl.) A. Gray aqueous extract compared to untreated hMSCs (control). AMPK is a key enzyme regulating several signals involved in metabolic pathways and appears to be intimately involved in adipocyte differentiation and maturation [63]; our result demonstrated that aqueous extract of leaves of *Tithonia diversifolia* (Hemsl.) A. Gray is able to inhibit adipocyte differentiation *in vitro* and its action involves pAMPK.

Presently intense interest by health care phytomedical reserch has focused on anti-inflammatory [2, 64, 65], antimalarial [66, 67], antidiabetic [68–70], and anticancer [71, 72] activities of *Tithonia diversifolia* (Hemsl.) A. Gray. Results obtained in the present research further support the use of *Tithonia diversifolia* (Hemsl.) A. Gray and its extracts in the therapy of several diseases and might offer new perspectives for therapeutic strategies in the treatment of obesity and related disorders.

## Conclusion

Results obtained in the present study confirmed antiradical capacity of *Tithonia diversifolia* (Hemsl.) A. Gray suggesting that its antioxidant action may be also due to the ability of affecting the expression of HO-1. In addition, the antiadipogenic activity of *Tithonia diversifolia* (Hemsl.) A. Gray found in hMSCs offer new stimulating perspective for future therapies, suggesting that its ability to inhibit adipocyte differentiation might be due to the activation of AMPK. Since activated AMPK regulates several metabolic pathways playing a pivotal role in the regulation of carbohydrate and fat metabolism, these findings might be usefull for the treatment of metabolic diseases such as diabetes and obesity. Results obtained in the present study also suggest that the intake of natural preparations of *T*. *diversifolia* might lower the risk of human diseases such as atherosclerosis, inflammation, ageing, ischemic reperfusion injury and neurodegenerative diseases.

## Supporting Information

S1 Fig Scavenger effect of extracts of *Tithonia diversifolia* (Hemsl.) A. Gray on superoxide anion expressed as percentage of inhibition of NADH oxidation (rate of superoxide anion production was 4 nmoles/min). (DOCX)

S2 Fig Effect of aqueous extract of *Tithonia diversifolia* (Hemsl.) A. Gray on LOOH production in plasma expressed as percentage of inhibition of LOOH formation with respect to the same sample incubated in absence of the extract. (DOCX)

S3 Fig Percentage of cell viability in hMSC cultures exposed to different concentrations of aqueous extract of leaves of *Tithonia diversifolia* (Hemsl.) A. Gray (DOCX)

S4 Fig Effect of aqueous extract of leaves of *Tithonia diversifolia* (Hemsl.) A. Gray on adipogenesis of hMSCs as measured by Oil-Red O staining (O.D. at λ = 490 nm). (DOCX)

S5 Fig Determination of ROS by DCFH method in cultured hMSC, Values represent Fluorescence Intensity (F.I.) x 1 x 10^6^/mg prot. (DOCX)

S6 Fig Protein expression of HO-1 and pAMPK by western blot analysis in cultured hMSCs; values are expressed as arbitrary units (A.U.). (DOCX)

S1 Table Total polyphenol content (expressed as μmM Gallic acid) and total flavonoid content (expressed as μmM Catechin) in three different extracts of leaves of *Tithonia diversifolia* (Hemsl.) A. Gray. (DOCX)

## In Memoriam

This work is dedicated to the memory of Prof. Liliana Iauk.
