# Supplementary material for: Effects of Tithonia diversifolia (Hemsl.) A. Gray Extract on Adipocyte Differentiation of Human Mesenchymal Stem Cells
Source: PLoS One. 2015 Apr 7;10(4):e0122320. doi: 10.1371/journal.pone.0122320 (PMC4388505; doi:10.1371/journal.pone.0122320)
Supplement: S1 Fig — (DOCX) [file pone.0122320.s001.docx]

**Individual data**

**Figure 1: SOD-like**

|  | **% inhibition of NADH oxidation** | **Means** | **S.D.** | **Medians** | **Variance measures** |
| --- | --- | --- | --- | --- | --- |
| Aqueous 0.087 μg/mL | 10.40 %  12.30 %  12.80 %  11.50 %  13.00 % | 11.88% | 0.879 | 12.2 | 0.77 |
| Aqueous 0.87 μg/mL | 28.00 %  30.00 %  31.00 %  32.00 %  33.00 % | 30.8% | 1.788 | 31 | 3.2 |
| Aqueous 4.44 μg/mL | 41.00 %  44.00 %  45.00 %  46.00 %  47.10 % | 44.62 % | 2.328 | 45 | 5.42 |
| Aqueous 17.5 μg/mL | 57.30 %  59.10 %  60.54 %  62.00 %  63.68 % | 60.52 % | 2.47 | 60.54 | 6.14 |
| Aqueous 44 μg/mL | 87.00 %  89.00 %  92.50 %  92.70 %  93.00 % | 90.8 % | 2.69 | 92.5 | 7.253 |
| Methanolic 0.087 μg/mL | 0.008 %  0.0009 %  0.00095 %  0.001 %  0.0015 % | 0.002 % | 0.003 | 0.001 | 9.61 E-8 |
| Methanolic 0.87 μg/mL | 5.10 %  4.94 %  4.85 %  5.12 %  5.00 % | 5.002 % | 0.11 | 5 | 0.012 |
| Methanolic 4.4 μg/mL | 21.05 %  21.10 %  20.86 %  20.94 %  21.06 % | 20.6 % | 1.78 | 21.5 | 3.17 |
| Methanolic 17.5 μg/mL | 42.00 %  43.30 %  44.10 %  44.30 %  48.30 % | 44.4 % | 2.36 | 44.10 | 5.57 |
| Methanolic 44 μg/mL | 65.00 %  66.00 %  67.00 %  68.00 %  72.00 % | 67.6 % | 2.7 | 67 | 7.3 |
| Dichloromethane 0.087 μg/mL | 0.0009 %  0.00093 %  0.0015 %  0.0089 %  0.0099 % | 0.0044% | 0.0045 | 0.0015 | 2.0799E-5 |
| Dichloromethane 0.87 μg/mL | 0.00089 %  0.00090 %  0.0012 %  0.0088 %  0.0099 % | 0.0043% | 0.0045 | 0.0012 | 2,11E-5 |
| Dichloromethane 4.4 μg/mL | 2.99 %  3.00 %  3.00 %  3.01 %  3.05 % | 3.01 % | 0.023 | 3 | 0.00054 |
| Dichloromethane 17.5 μg/mL | 2.98 %  3.00 %  3.00 %  3.05 %  3.01 % | 3.008 % | 0.0258 | 3 | 0.0006 |
| Dichloromethane 44 μg/mL | 19.88 %  19.99 %  20.03 %  20.05 %  20.10 % | 20.01 % | 0.082 | 20.03 | 0.0068 |
| SOD 80 mU | 89 %  90 %  91 %  90 %  92 % | 90. 4 % | 1.14 | 90 | 1.299 |
