# Supplementary material for: Effects of Tithonia diversifolia (Hemsl.) A. Gray Extract on Adipocyte Differentiation of Human Mesenchymal Stem Cells
Source: PLoS One. 2015 Apr 7;10(4):e0122320. doi: 10.1371/journal.pone.0122320 (PMC4388505; doi:10.1371/journal.pone.0122320)
Supplement: S2 Fig — (DOCX) [file pone.0122320.s002.docx]

**Individual data**

**Figure 2: LOOH**

|  | **% inhibition of LOOH** | **Means** | **S.D.** | **Medians** | **Variance measures** |
| --- | --- | --- | --- | --- | --- |
| Aqueous 0.0087 μg/mL | 18.00%  18.00%  18.10%  18.30%  18.40% | 18.16 % | 0.18 | 18.1 | 0.0329 |
| Aqueous 0.087 μg/mL | 38.95 %  39.00 %  40.50 %  41.86 %  43.00 % | 40.66 % | 1.77 | 40.5 | 3.55 |
| Aqueous 0.87 μg/mL | 59.80 %  63.00 %  64.00 %  64.00 %  66.00 % | 63.36 % | 2.26 | 64 | 5.148 |
| Aqueous 8.7 μg/mL | 67.00 %  70.95 %  71.00 %  71.00 %  73.00 % | 70.59 % | 2.188 | 71 | 4.79 |
| Aqueous 17.5 μg/mL | 78.00 %  79.00 %  81.00 %  82.00 %  83.00 % | 80.6 % | 2.073 | 81 | 4.3 |
| Aqueous 44 μg/mL | 95.60 %  99.00 %  99.20 %  99.85 %  100 % | 98.73 % | 1.799 | 99.20 | 3.23 |
