# Supplementary material for: Effects of Tithonia diversifolia (Hemsl.) A. Gray Extract on Adipocyte Differentiation of Human Mesenchymal Stem Cells
Source: PLoS One. 2015 Apr 7;10(4):e0122320. doi: 10.1371/journal.pone.0122320 (PMC4388505; doi:10.1371/journal.pone.0122320)
Supplement: S3 Fig — (DOCX) [file pone.0122320.s003.docx]

**Individual data**

**Figure 3: MTT ASSAY**

|  | **% cell viability** | **Means** | **S.D.** | **Medians** | **Variance measures** |
| --- | --- | --- | --- | --- | --- |
| Control | 96.00 %  96.80 %  99.00 %  99.00 %  100.00 % | 98.16 % | 1.68 | 99 | 2.82 |
| Aqueous 0.087 μg/mL | 95.00 %  97.00 %  98.00 %  99.00 %  100.00 % | 97.8 % | 1.923 | 98 | 3.70 |
| Aqueous 0.87 μg/mL | 95,00 %  97,00 %  97,00 %  98,00 %  99,00 % | 97.2 % | 1.483 | 97 | 2.2 |
| Aqueous 4.4 μg/mL | 95.00 %  97.00 %  97.00 %  99.00 %  99.00 % | 97.4 % | 1.67 | 97 | 2.799 |
| Aqueous 17.5 μg/mL | 95.00 %  96.00 %  97.00 %  99.00 %  99.00 % | 97.2 % | 1.78 | 97 | 3.20 |
| Aqueous 44 μg/mL | 96.00 %  97.00 %  99.00 %  100.00 %  100.00 % | 98.4 % | 1.81 | 99 | 3.29 |
| Aqueous 175 μg/mL | 95.00 %  98.00 %  99.00 %  99.00 %  100.00 % | 98.2 % | 1.92 | 99 | 3.70 |
