# Supplementary material for: Effects of Tithonia diversifolia (Hemsl.) A. Gray Extract on Adipocyte Differentiation of Human Mesenchymal Stem Cells
Source: PLoS One. 2015 Apr 7;10(4):e0122320. doi: 10.1371/journal.pone.0122320 (PMC4388505; doi:10.1371/journal.pone.0122320)
Supplement: S4 Fig — (DOCX) [file pone.0122320.s004.docx]

**Individual data**

**Figure 4: Oil-Red O Staining**

|  | **Oil-Red O Staining O.D. at λ=490 nm** | **Means** | **S.D.** | **Medians** | **Variance measures** |
| --- | --- | --- | --- | --- | --- |
| MSC control | 0.31  0.32  0.32  0.33  0.34 | 0.324 | 0.011 | 0.32 | 0.0001 |
| Aqueous 17.5 μg/mL | 0.26  0.28  0.30  0.31  0.32 | 0.294 | 0.024 | 0.3 | 0.00057 |
| Aqueous 175 μg/mL | 0.22  0.27  0.28  0.30  0.32 | 0.278 | 0.037 | 0.28 | 0.00142 |
