# Supplementary material for: Effects of Tithonia diversifolia (Hemsl.) A. Gray Extract on Adipocyte Differentiation of Human Mesenchymal Stem Cells
Source: PLoS One. 2015 Apr 7;10(4):e0122320. doi: 10.1371/journal.pone.0122320 (PMC4388505; doi:10.1371/journal.pone.0122320)
Supplement: S5 Fig — (DOCX) [file pone.0122320.s005.docx]

**Individual data**

**Figure 5: ROS**

|  | **F.I.** | **Means** | **S.D.** | **Medians** | **Variance measures** |
| --- | --- | --- | --- | --- | --- |
| MSC control | 30.00  32.00  33.00  34.00  35.00 | 32.8 | 1.923 | 33 | 3.7 |
| Aqueous 17.5 μg/mL | 15.00  16.00  17.00  18.00  20.00 | 17.2 | 1.92 | 17 | 3.7 |
| Aqueous 175 μg/mL | 10.00  10.00  11.00  12.30  14.00 | 11.46 | 1.70 | 11 | 2.908 |
