# Supplementary material for: Effects of Tithonia diversifolia (Hemsl.) A. Gray Extract on Adipocyte Differentiation of Human Mesenchymal Stem Cells
Source: PLoS One. 2015 Apr 7;10(4):e0122320. doi: 10.1371/journal.pone.0122320 (PMC4388505; doi:10.1371/journal.pone.0122320)
Supplement: S6 Fig — (DOCX) [file pone.0122320.s006.docx]

**Individual data**

**Figure 6: Western blot**

|  | **A.U. for HO-1** | **Means** | **S.D.** | **Medians** | **Variance measures** |
| --- | --- | --- | --- | --- | --- |
| hMSC control | 0.5263  0.5892  0.5992  0.6466  0.7177 | 0.6158 | 0.0712 | 0.5992 | 0.0050 |
| Aqueous 175 μg/mL | 1.1666  1.1748  1.1810  1.1820  1.2816 | 1.1972 | 0.0475 | 1.1810 | 0.0022 |
|  |  |  |  |  |  |
|  | **A.U. for pAMPK** | **Means** | **S.D.** | **Medians** | **Variance measures** |
| hMSC control | 0.6301  0.8312  0.7525  0.7221  0.8458 | 0.7563 | 0.0876 | 0.7525 | 0.00768 |
| Aqueous 175 μg/mL | 0.9725  0,9920  0.9258  0.9512  1.0100 | 0.9703 | 0.0331 | 0.9725 | 0.00109 |
