# Supplementary material for: Effects of Tithonia diversifolia (Hemsl.) A. Gray Extract on Adipocyte Differentiation of Human Mesenchymal Stem Cells
Source: PLoS One. 2015 Apr 7;10(4):e0122320. doi: 10.1371/journal.pone.0122320 (PMC4388505; doi:10.1371/journal.pone.0122320)
Supplement: S1 Table — (DOCX) [file pone.0122320.s007.docx]

**Individual data**

**Table 1: Total Phenolic content in three different extracts of leaves of *Titonia diversifolia* (Hemsl.) A. Gray**

| **Extract** | **μM Gallic acid** | **Means** | **S.D.** | **Medians** | **Variance measures** |
| --- | --- | --- | --- | --- | --- |
| Aqueous | 51.88  51.92  51.99  52.04  52.08 | 51.982 | 0.08 | 51.99 | 0.0068 |
| Methanolic | 30.91  30.923  30.945  31.09  31.09 | 30.99 | 0.09 | 30.945 | 0.0082 |
| Dichloromethane | 28.98  28.98  29.02  29.02  29.02 | 29.04 | 0.02 | 29.02 | 0.00047 |

**Table 1: Total Flavonoid content in three different extracts of leaves of *Titonia diversifolia* (Hemsl.) A. Gray**

| **Extract** | **μM Catechin** | **Means** | **S.D.** | **Medians** | **Variance measures** |
| --- | --- | --- | --- | --- | --- |
| Aqueous | 58.91  58.91  59.03  59.09  59.09 | 59.006 | 0.09 | 59.03 | 0.00828 |
| Methanolic | 33.93  33.93  34.02  34.07  34.07 | 34.004 | 0.07 | 34.02 | 0.00498 |
| Dichloromethane | 38.94  38.94  38.98  39.06  39.06 | 38.996 | 0.06 | 38.98 | 0.0036 |
